# Epidemiological Trends and Clinical Implications: An 8-Year Retrospective Study from a Burn Rehabilitation Center in Taiwan

**DOI:** 10.3390/medicina62081494

**Published:** 2026-08-03

**Authors:** Chih-Kuan Wu, Shih-Yi Yang, Ju-Wen Cheng, Chien-Hung Chen, Pei-Hsuan Wu

**Affiliations:** 1 Department of Physical Medicine and Rehabilitation, Linkou Chang Gung Memorial Hospital, Taoyuan 33305, Taiwan; xavierwu829@gmail.com (C.-K.W.); b9002052@cgmh.org.tw (C.-H.C.); 2 School of Physical Therapy and Graduate Institute of Rehabilitation Science, Chang Gung University, Taoyuan 33302, Taiwan; 3 Center of Comprehensive Sports Medicine, Taoyuan Chang Gung Memorial Hospital, Taoyuan 33378, Taiwan; 4 Department of Plastic and Reconstructive Surgery, Burn Center, Linkou Chang Gung Memorial Hospital, Taoyuan 33305, Taiwan; yangshihyi@gmail.com; 5 College of Medicine, Chang Gung University, Taoyuan 33302, Taiwan; 6 Burn Rehabilitation Center, Taoyuan Chang Gung Memorial Hospital, Taoyuan 33378, Taiwan; 7 Department of Physical Medicine and Rehabilitation, Taoyuan Chang Gung Memorial Hospital, Taoyuan 33378, Taiwan; sophie1976@gmail.com

**Keywords:** epidemiology, burn, occupational injury, rehabilitation

## Abstract

*Background and Objectives*: Advances in medical, surgical, and acute burn care have shifted burn care from survival alone to survivorship; such improvement can be accompanied by extensive morbidity and disability in chronicity not previously encountered. The epidemiological characteristics of patients treated at a burn rehabilitation center, in the context of evolving social and occupational environments, may provide insights into current trends and their clinical implications. Notably, updated epidemiological data reflecting these changes in burn injury patterns remain scarce worldwide. To date, no epidemiological data from a burn rehabilitation center have been reported in Taiwan. Therefore, this study aimed to bridge this gap by providing a recent epidemiological review from the perspective of a burn rehabilitation center. *Materials and Methods*: This is a single-center, retrospective cross-sectional study enrolling patients who sustained burn injuries between January 2016 and December 2023. Data were collected from medical records and analyzed using SPSS version 20.0. *Results*: A total of 143 patients were recruited, of whom 101 were male (70.6%). The mean age was 38.51 years (SD = 20.28), and the mean total body surface area (TBSA) burned was 20.83% (SD = 18.77). Flame burns were the most common etiology, accounting for 67 cases (46.9%). Among the 143 patients, 73 (51.0%) sustained work-related burns, which were associated with a higher incidence of eye involvement compared with non-work-related burns (28.8% vs. 14.3%, *p* = 0.036). The upper extremity was the most frequently involved anatomical site, affecting 98 patients (68.5%). *Conclusions*: The present findings highlight an underrecognized need for stronger safety measures that address both personal and environmental risk factors. Risk-reduction strategies should particularly account for the aging workforce and vulnerable anatomical sites to mitigate preventable human and economic losses.

## 1. Introduction

Burns remain a major cause of trauma worldwide, prevalent in both developed and underdeveloped countries [1]. Burn injuries not only result in mortality and morbidity, but also lead to post-burn complications, scar contractures, pain, and pruritus, profoundly affecting the survivors’ function, quality of life, and the economy of the nation.

Burn survivors often struggle with long-lasting physical and psychosocial issues. Consequently, surgical intervention, active rehabilitation, and psychological support are warranted, imposing a considerable financial burden [2]. Undoubtedly, prevention stands as the primary treatment for burns. Moreover, effective burn prevention strategies necessitate reliable knowledge on epidemiological features across timeframe and population characteristics. It is noteworthy that the most up-to-date epidemiological studies of burns in Taiwan were cohort reports in 2010 and 2013, respectively [3,4].

Advancements and innovations in resuscitation, critical care, antibiotic administration, and surgical techniques have led to a higher survival rate among individuals with major burns, consequently increasing the demand for subsequent rehabilitation. The feasibility and effectiveness of developing a burn rehabilitation unit were first introduced in 1998 [5]. Moreover, Sliwa et al. [6] presented the epidemiology of survivors receiving inpatient rehabilitation at a single burn rehabilitation center. Additionally, two multi-center studies were conducted to explore comprehensive post-burn rehabilitation outcomes in the United States [7,8], highlighting the significance of post-burn rehabilitation and the rarity of such research. To our knowledge, no prior study in Taiwan has detailed the epidemiology of a hospital-based burn rehabilitation center. This study aims to identify burn patterns, assess rehabilitation needs, and explore preventive measures. Furthermore, by examining the epidemiology of the burn rehabilitation center, we look forward to identifying societal trends and demands for post-burn rehabilitation, which consequently enables us to optimize resource allocation while remaining sensitive to social changes and other developments.

## 2. Materials and Methods

### 2.1. Study Design, Settings, and Population

Taiwan’s largest burn center was established in 1978 at Chang Gung Memorial Hospital (CGMH) [9]. It is a major regional referral center that admits more than 300 patients annually and primarily serves northern Taiwan. The center has provided resuscitation and long-term care for some of the most medically complex burn cases in Taiwan and is widely recognized for its active role in managing mass burn casualties following the colored powder explosion on 27 June 2015 [10]. To meet the unprecedented demand for burn rehabilitation after this mass casualty event [11], an outpatient burn rehabilitation center was established in 2015. A burn rehabilitation center is distinct from an acute burn center, as it primarily manages patients who are medically stable and no longer require acute care but continue to experience persistent post-burn impairments. At this stage, the primary focus of medical care shifts to restoring range of motion, muscle strength, and endurance, as well as scar management. This multidisciplinary center met the criteria proposed by DeSanti et al. [5] for a level I burn rehabilitation unit, including the admission of more than 20 new patients annually. Approximately 80% of patients were discharged from the local burn center, with additional referrals from other regions in Taiwan.

This retrospective cross-sectional study analyzed post-burn patients at the CGMH burn rehabilitation center from January 2016 to December 2023. Patients with incomplete burn data were excluded. Chart reviews via the hospital information system (HIS) included medical, nursing, and clinical records. Data retrieved covered demographics (sex, age, ethnicity, literacy), burn characteristics (onset date, season, time, activity, location, etiology, TBSA, depth), and clinical outcomes (inhalation injury, ventilator use, hospital stay, surgical details, and physiatrist consultation dates).

### 2.2. Statistical Analysis

Statistical analysis was performed using the IBM SPSS (version 20.0 for windows; Chicago, IL, USA). Descriptive statistics were presented by mean and standard deviation (SD) or number and percentage. Chi-square and Fisher’s exact *t*-test were utilized for categorical variables, and Student’s *t*-test or one-way analysis of variance for continuous variables when appropriate, with a *p*-value lower than 0.05 considered statistically significant.

### 2.3. Ethical Consideration

Ethical approval was obtained from the Institutional Review Board of Chang Gung Memorial Hospital with the approval number (202400329B0), approval date: 11 March 2024. Patient informed consent was waived due to the anonymous nature of the data collected. No patients or members of the public were involved in the design, conduct, reporting, or dissemination of this study. This study used secondary data and therefore did not require direct patient or public involvement.

## 3. Results

### 3.1. Age, Sex, Ethnicity

A total of 143 burn survivors were collected in our burn rehab center over an 8-year period. Table 1 demonstrates the demographics of the patients. The mean age of the patients was 38.51 (SD = 20.28) years, ranging from 0 to 83 years. Among them, 22 individuals (15.4%) were children under the age of 18, with the mean age of 3.81 (SD = 3.61) years. The mean age of the adult patients was 44.82 (SD = 14.95) years. The two predominant age groups were between 19 and 40 years (N = 50, 35.0%) and between 41 and 60 years (N = 52, 36.4%). The overall male to female ratio was 2.40:1 (101 males to 42 females), while in the children’s group, it was 0.83:1 (10 males to 12 females). The majority of our study population was Taiwanese, with 11.8% (N = 17) being foreigners from Vietnam, Indonesia, the Philippines, and Thailand.

### 3.2. Education Level

The majority of the burn survivors (N = 106, 86.2%) attained at least a primary school level of schooling, more than half of the patients (N = 79, 64.3%) completed senior high school or higher education. Nonetheless, up to 13.8% (N = 17) of the patients were illiterate with limited education. (Table 1).

### 3.3. Seasonal Variation and Time of Day

All seasons were vulnerable to burn injuries with an increasing trend during spring (N = 40, 28.0%) here in our burn rehabilitation center, followed by winter (N = 35, 24.5%), summer (N = 34, 23.8%) and autumn (N = 34, 23.8%). Moreover, there was a higher prevalence of electrical burns (N = 4, 36%) and flame burns caused by wiring faults and utility box explosions (N = 7, 43.8%) in spring. In terms of the time of day, the majority of burn injuries occurred in the afternoon, between 13:00 and 17:00 (N = 43, 30.1%), with increased incidences between 09:00 and 13:00 (N = 37, 25.9%) and 17:00 to 21:00 (N = 32, 22.4%).

### 3.4. Etiology of Burns

Table 2 showed the burn features of the study population. Flame including flashes and explosions was the leading etiology of burns, accounting for 46.9% (N = 67) of the burn injuries, followed by scalds (N = 38, 26.6%) and contact burns (N = 16, 11.2%). On the other hand, scalds and contact burns accounted for 81.8% (N = 18) of the pediatric burn population.

### 3.5. Activity When Burn Occurreds

**Figure 1:**
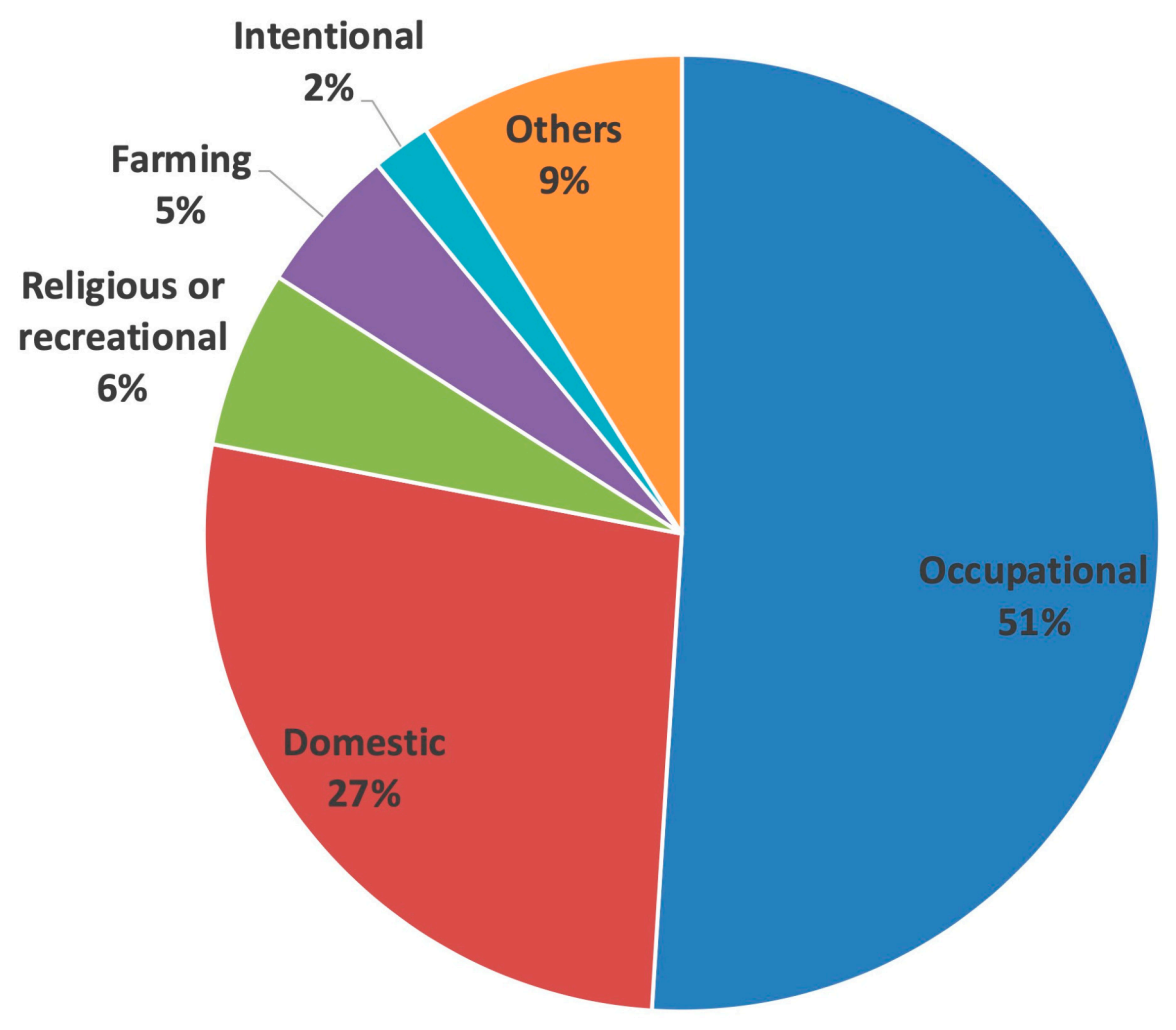
Demographic distribution of burn etiologies at the burn rehabilitation center. The activities when burn injuries occurred: occupational: 51%; domestic: 27%; others: 9%; religious or recreational: 6%; farming: 5%; intentional: 2%.

Over half of the burn survivors (N = 73, 51.0%) were at work when the burns occurred. In addition, 27.3% (N = 40) of the burns happened at place of residence (Figure 1). On the other hand, 16.8% (N = 24) of the patients were handling meals during the burn insult. Remarkably, there were five cases (3.5%) of injury by firecrackers at religious events and three cases (2.1%) of injury during farming or hay burning.

### 3.6. Severity and Depth of Burns

The overall mean TBSA was 20.83% (SD = 18.77) with a range of 0.5 to 85%. Notably, 44.06% (N = 63) of the burn survivors experienced major burns with TBSA ≥ 20% (Table 2). According to the etiology, flame burns had significantly higher mean TBSA (26.75%, *p* < 0.001) compared to non-flame burns (15.62%). The mean TBSA for adults was 21.24%, which did not differ significantly from the TBSA for children (18.61%). No significant differences were noted in TBSA between the work- and the non-work-related groups. In addition, 79.7% (N = 114) of the burns had a burn depth of 2 to 3 degrees.

### 3.7. Anatomical Location of Burns

The upper extremity (N = 98, 68.5%) was the most frequently affected burn site, followed by hand (N = 72, 50.3%), trunk (N = 68, 47.6%), and lower extremity (N = 66, 46.2%), as shown in Table 2. Notably, 84.6% (N = 121) of burns involved multiple sites, defined as injuries occurring in at least two distinct anatomical sites such as head and neck, upper limb, trunk, lower limb, or perineum. In flame burns, involvement of the facial region including the eye and the neck with the upper extremity was significantly more common (*p* < 0.05) compared to non-flame burns. Such manifestations are nearly duplicated in work-related burns. Involved anatomical sites such as the eye, the face, and the upper extremity (*p* < 0.05) are all similar in presentation, while the foot was less frequently involved. Table 3 demonstrates the susceptibility of anatomical sites to work-related burn injuries compared to the study conducted by McInnes et al. [12]. In contrast, scald burns were associated with less involvement of the facial region and the hand (*p* < 0.05).

### 3.8. Clinical Course During the Preceding Acute-Care Hospitalization

To characterize the clinical severity of the study population before rehabilitation, we examined their medical records from the preceding acute-care hospitalization. Among the 143 patients subsequently treated at the burn rehabilitation center, 129 patients had previously been admitted to an acute burn center, 31.8% of them had mechanical ventilator-support, the mean duration of mechanical ventilator use was 9.12 (SD = 6.43) days (Table 4). In addition, 22.4% (N = 29) of the burn patients were diagnosed with inhalational injury via bronchoscopy evaluation. The mean length of hospital stay was 26.98 (SD = 15.75) days. 86% (N = 100) of the admissions received surgical treatments, with 62.6% (N = 72) undergoing skin grafts. The mean number of surgeries performed during admission was 2.97. The mean time to first debridement was 3.31 (SD = 2.33) days, while the mean time to first skin graft was 10.57 (SD = 3.10) days. Furthermore, the mean time to first physiatrist consultation was 3.74 (SD = 2.81) days.

## 4. Discussion

### 4.1. Age, Sex, Ethnicity

The overall mean age of our population was similar to the studies from Germany (41.6) [13], Catalonia (41.4) [14], Singapore (42.6) [15], and South Korea (39.9) [16], but higher than the mean ages from the US (32) [17], Australian & New Zealand Burn Association (ANZBA) (30) [18] and developing countries [19,20,21]. It is noteworthy that a study conducted in Tokyo [22] reported the oldest mean age of 50 worldwide, which correlated with the high life expectancy of 85 years in Japan in 2020 [23]. In comparison to the earlier studies in Taiwan [3,4,24,25], our research revealed an escalating trend of mean age from 29 to 38.5 years; such changes reflect the global trend of aging populations. The observed trend of older mean age among adult burn survivors was consistent with findings from various articles [4,12,14,16,22], indicating an aging trend within the working population. Age-related declines in vision, hearing, and response time underscore the need for age-appropriate workplace safety adaptations. Men were the predominant gender in burn cases, which is consistent with studies across regions [4,12,14,15,16,22,25,26,27], likely due to men’s greater involvement in high-risk industries. Exceptions include Manipal [19], and outpatient-based studies from South Korea [28] and Taiwan [3]. Over 10% of cases involved foreigners, highlighting the need to address language and cultural barriers through staff education, given Taiwan’s growing immigrant labor force.

### 4.2. Education Level

Given the high level of literacy in Taiwan compared to Bangladesh and India [19,20], the risk of accidental burns and minor burn injuries could be mitigated through comprehensive public education initiatives. Additionally, survivors with higher levels of education tend to acknowledge the significance of post-burn rehabilitation, resulting in increased motivation and adherence to treatment programs [29]. Further research is warranted to clarify the relation between education level and compliance to rehabilitation.

### 4.3. Etiology of Burns

The shift from scald to flame burns aligns with trends in developed countries [12,14,15,16,22,25]. In northern Taiwan, flame burns dominate, especially in industry, highlighting the need for updated safety policies and proper storage of flammable materials. Scald burns remain predominant in children (54.5%), which is consistent with other studies [14,16,25], suggesting preventability through parental education and keeping hot items out of reach.

### 4.4. Activity When Burns Occurred

Half of our study population sustained work-related injuries, a notably higher proportion than the 15–30% reported in both developed and developing countries [12,13,21,22,26,30] and the 32.1% found by Tung et al. [25]. This high incidence may be influenced by our center’s geographical location and the referral characteristics of the burn rehabilitation patients. Domestic burn survivors, often with minor scald or contact burns, typically receive outpatient treatment and engage in home-based exercises [3]. In contrast, work-related burn survivors, primarily injured by flame, experience greater TBSA, scar contracture, functional disability, and impaired self-image. As key financial contributors, they are more motivated to undergo rehabilitation to regain function which enables an earlier possible return to work. This underscores the necessity of post-burn rehabilitation and resource allocation for work retraining and adaptation programs.

### 4.5. Severity and Depth of Burns

Despite Taiwan’s advanced medical system, our study demonstrated a high mean TBSA and severity, aligning with findings from developing countries [20,21,26] rather than developed nations (4–14%) [12,14,15,22,31]. As Northern Taiwan’s main burn referral center, the CGMH burn rehabilitation center receives more cases of major burns, while minor cases are managed locally. The characteristics of rehabilitation patients further influenced burn severity analysis. The disparity between the high level of development and the great severity of burns implies the persistent prevalence of major burns and the preventability of minor burns. Considering the unpredictability of massive burn tragedies and the temporal neutrality in the occurrence of major burns, having a burn intensive care unit equipped with a multi-disciplinary team is crucial.

### 4.6. Anatomical Location of Burns

Upper extremities and hands were frequent sites of burn involvement. This is consistent with several studies, reporting about 40% [3,12,14], probably because of the active role of the upper limbs in task execution, as well as their function as protective shields for the face during reflex reactions to burns. Given that hands and upper extremities are vital for performing activities of daily living and work tasks, and that as demonstrated by Hartl et al. [32], they account for most of the body’s cutaneous functional units, they often require a considerable amount of therapy time. Therefore, attention should be given to resource allocation for targeted anatomical sites to ensure that active rehabilitation is well-planned and designed for timely recovery. Trunk involvement is also common in severe burns, necessitating stability, endurance, and strength training for posture and energy efficiency. Most patients had multiple anatomical sites affected, complicating rehabilitation due to scar-induced soft tissue immobility, myofascial pain [2], and restricted joint flexibility and kinetic chains.

The frequent involvement of the face in burn injuries, as reported by Yamamoto et al. (31.7%) [22] and McInnes et al. (28.9%) [12], underscores the distressing impact on social and financial burdens. Facial burns not only cause disfigurement and diminish self-esteem but they may affect the five senses of the face in varying degrees: namely ophthalmoception, audioception, gustacoception, olfacoception, and tactioception. Challenges of facial burn survivors were outlined [33], such as body suffering, identity reset, cultural stigma, and perceived social rejection, imposing the need for physical and psychosocial rehabilitation. Scar contracture around the mouth, neck, or chin can restrict mouth opening, alter facial expression, and cause dysphagia, poor oral hygiene, and impaired social interaction [34], highlighting the need for targeted interventions.

The ignitive nature of flashes and explosions showed that the incidence of eye thermal injuries (28.8%) in occupational accidents was significantly higher than the 5.5% reported by McInnes et al. [12], likely due to flashes and explosions in occupational accidents. Facial protection should be prioritized when handling flammable materials. Table 3 shows a higher proportion of face and upper extremity involvement in both work- and non-work-related burns compared to ANZBA data, underscoring the need for enhanced labor safety and tailored rehabilitation. Notably, flame burns often spread in an anti-gravity pattern from hands to upper extremities, neck, and face, a pattern not observed in other etiologies. In terms of the universal similarity of the most common etiology of flame burns among burn rehab centers worldwide [7,8] and the prevalence of occupation-related burns in this current study, efforts and resources should be focused on targeted anatomical sites most vulnerable to burn injuries and their consequences.

### 4.7. Clinical Outcomes During Admission

The high rate of inhalational injury in this study surpassed most regional reports [14,15,17,24,26,27], except Tokyo (42%) [22] and South Western Nigeria (32%) [35], likely due to the high proportion of flame burns. Our mean hospital stay exceeded those in developed countries and prior Taiwanese studies [14,15,17,22,24,26,27], possibly due to greater burn severity and surgical needs, as noted by Kruger et al. [36]. Surgical rate, number of surgeries, and skin graft proportion were also higher than in other studies [12,14,15,24,31,37], reflecting the implementation of early debridement, grafting, and timely physiatrist consultation. Given risks of post-burn disability and graft-related complications, early, individualized rehabilitation during the acute phase is essential. Subacute rehabilitation remains crucial, especially for injuries involving functional sites such as the hand, eye, ear, and genital areas.

### 4.8. Epidemiological Data of Burn Rehabilitation Taiwan

Table 5 presents epidemiological data from 1996 to 2023 in Taiwan, encompassing various age groups and time periods, with data sources ranging from single-center studies to nationwide population-based analyses [3,24,38]. Three major trends were identified: (1) the predominant injury site shifted from the trunk and lower extremities to the upper extremities and hands among adults; (2) the leading cause of burns changed from scalds to flames; and (3) the mean patients age increased from 29.2 to 38.5 years. These findings highlight the importance of protective equipment and an aging workforce, emphasizing the key role of rehabilitation centers in reducing burn-related impairments.

### 4.9. Strengths and Limitations

This study has several strengths and limitations. First, it was conducted at a single burn rehabilitation center, which may limit the generalizability of the findings to the national population. In addition, the center is located in an industrial region, resulting in a predominance of male patients and work-related burn injuries; therefore, caution is warranted when interpreting the findings. Furthermore, outcome data were not available in the current dataset, and future studies are needed to evaluate long-term outcomes and the effectiveness of rehabilitation interventions. Nevertheless, to our knowledge, this is the first study to report the epidemiology of patients receiving outpatient burn rehabilitation in Taiwan, filling an important gap in the current literature and providing contemporary evidence to better characterize this patient population.

## 5. Conclusions

This retrospective study provides epidemiological insights from a burn rehabilitation center. Cultural, occupational, and environmental factors appear to substantially influence burn mechanisms, suggesting the need for region-specific and context-sensitive prevention strategies. In rapidly evolving industrialized societies, burn rehabilitation centers may serve as valuable sources of epidemiological surveillance for identifying underrecognized safety concerns and emerging patterns of burn injuries. These findings may help inform ongoing monitoring, preventive strategies, and the continued development of comprehensive burn rehabilitation systems.

## Figures and Tables

**Table 1:** Demographic characteristics of the burn survivors.

Variables	N	%
Age (years)	38.51 ^a^ ± 20.28 (0~83)	-
0~18	22	15.4
19~40	50	35.0
41~60	52	36.4
61~80	17	11.9
81~99	2	1.4
Total	143	100
Adult mean age	44.82 ^a^ ± 14.95 (19~83)	-
Pediatric mean age	3.81 ± 3.61 (0~14)	-
Sex		
Male	101	70.6 ^a^
Female	42	29.4
Ethnicity		
Taiwanese	126	88.1
Non-Taiwanese	17	11.9
Education level		
Illiteracy	17	13.8
Primary	17	13.8
Junior high	10	8.1
Senior high	43	35.0
College	36	29.3
Incomplete data	20	-
Effective samples	123	100

Descriptive statistics are presented as mean ± standard deviation. ^a^ The finding showed similar presentation to other studies.

**Table 2:** Burn features of the burn survivors.

Variables	N	%
Mechanism/Etiology		
Flame	67	46.9 ^a^
Scald	38	26.6
Electrical	11	7.7
Contact	16	11.2
Chemical	9	6.3
Others	2	1.4
Total	143	100
TBSA (%)	20.83 ^b^ ± 18.77 (0.5~85)	-
0~19	80	56.0
20~39	42	29.4
>40	21	14.6
Total	143	100
Adult TBSA	21.24 ± 18.76 (0.5~85)	-
Children TBSA	18.61 ± 19.54 (0.5~70)	-
Burn degree		
2~3	114	79.7
Total	143	100
Anatomical sites		
Face	62	43.4
Neck	40	27.8
Trunk	68	47.6
Upper extremity	98	68.5
Hand	72	50.3
Lower extremity	66	46.2
Foot	23	16.1
Multiple sites (≥2 anatomical sites)	121	84.6

Descriptive statistics are presented as mean ± standard deviation. ^a^ The finding showed a presentation similar to that of other studies. ^b^ The finding showed a higher percentage compared to other studies. TBSA: Total body surface area.

**Table 3:** The susceptibility of anatomical sites to work-related burn injuries.

	12 Burn Centers, ANZBA, 2009~2016			Burn Rehabilitation Center, Northern Taiwan, 2016~2023		
Anatomical Sites	Work (*n* = 1828)	Non-Work (*n* = 8746)	*p*-Value ^a^	Work (*n* = 73)	Non-Work (*n* = 70)	*p*-Value ^a^
Eye	5.5%	3.1%	<0.001 *	28.8%	14.3%	0.036 *
Face	30.3%	28.9%	0.250	53.4%	32.9%	0.013 *
Neck	14.1%	13.5%	0.517	34.2%	21.4%	0.088
Trunk	17.2%	22.8%	<0.001 *	49.3%	45.7%	0.666
Upper extremity	42.2%	42.0%	0.871	78.1%	58.6%	0.012 *
Hand	36.0%	37.6%	0.444	53.4%	42.9%	0.453
Lower extremity	33.8%	44.0%	0.001 *	50.0%	42.5%	0.366
Foot	17.8%	16.6%	0.001 *	9.6%	22.9%	0.031 *

ANZBA: The Australian & New Zealand Burn Association; * *p* < 0.05; ^a^ Chi-square test was used to calculate *p* value.

**Table 4:** Clinical characteristics and treatment course during the preceding acute-care hospitalization among patients subsequently admitted to the burn rehabilitation center (N = 129).

Variables	N	%
Inhalational injury	29	22.4
Duration of mechanical ventilator use (days)	9.12 ± 6.43 (1~30) ^a^	-
Hospital stay (days)	26.98 ± 15.75 (3~98) ^a^	-
Surgery number during admission	2.97 ± 2.15 (1~13) ^a^	-
Time to first debridement (days)	3.31 ± 2.33 (0~15)	-
Time to first grafting (days)	10.57 ± 3.10 (4~19)	-
Time to first physiatrist consultation (days)	3.74 ± 2.81 (1–17)	-

Descriptive statistics are presented as mean ± standard deviation. ^a^ The finding showed a higher percentage compared to other studies.

**Table 5:** Epidemiological data of burn rehabilitation Taiwan.

	Southern Taiwan	Taiwan	Taiwan	Northern Taiwan
Date source	Single center ^†^	BIIS ^‡^	NHIRD ^§^	Single center
Study period	1996~2002	1997~1999	2010	2016~2023
Patient number	157	4741	7630	143
Patient/center/year	22.4	69.7	NA	17.9
Patient selection	Inpatient	Inpatient	Inpatient/Outpatient	Outpatient
Mean age (years)	4.03	29.2	NA	38.5
Gender (M/F)	52.2/47.8	67.0/33.0	43.3/56.7	70.6/29.4
Etiology	Scald (75.2%) Flame (22.3%)	Scald (49.9%) Flame (19.6%)	NA	Flame (47%) Scald (26.6%)
Mean TBSA	15.4%	15.0%	NA	20.8%
Site of injury				
Head	68.2%	11.6%	16.7%	43.4%
Eye	NA	0.8%	NA	NA
Neck	NA	7.1%	NA	27.8%
Trunk	78.6%	19.4%	7.5%	47.6%
Upper extremity	60.3%	17.8%	40.4%	68.5%
Hand	NA	14.6	NA	50.3%
Lower extremity	42.9%	18.7	32.0%	46.2%
Feet	NA	7.8%	NA	16.1%
Genitalia	18.6%	1.7	NA	NA

NA: Not applicable. ^†^ Data from Kaohsiung Medical University Hospital (single-center retrospective study). ^‡^ Data from the Burn Injury Information System (BIIS; multi-center registry). ^§^ National Health Insurance Research Database (NHIRD; nationwide population-based).

## Data Availability

All data analyzed during the current study are available from the corresponding author on reasonable request.

## Abbreviations

The following abbreviations are used in this manuscript:

CGMH	Chang Gung Memorial Hospital
TBSA	Total body Surface Area
SD	Standard Deviation
ANZBA	Australian & New Zealand Burn Association
BIIS	Burn Injury Information System
NHIRD	National Health Insurance Research Database
